# LncRNA6470/ace-miR-750-y axis modulates *AcPP2A* gene, immune response and survival of eastern honey bee larvae during fungal infection

**DOI:** 10.1080/21505594.2026.2697761

**Published:** 2026-08-02

**Authors:** Xiaoxue Fan, Kaiyao Zhang, Yunzhen Yang, Xinrui Chen, Wei Wang, Siyi Wang, Jianfeng Qiu, Qingwei Tan, Dafu Chen, Rui Guo

**Affiliations:** a College of Bee Science, Fujian Agriculture and Forestry University, Fuzhou, China; b National & Local United Engineering Laboratory of Natural Biotoxin, Fuzhou, China; c Apitherapy Research Institute of Fujian Agriculture and Forestry University, Fuzhou, China

**Keywords:** *Apis cerana*, *Ascosphaera apis*, immune response, ace-miR-750-y, *AcPP2A*, competing endogenous RNA

## Abstract

Chalkbrood, a destructive larval disease caused by the fungal pathogen *Ascosphaera apis*, causes substantial losses in apiculture. Although host–pathogen interactions in *Apis mellifera* larvae infected with *A. apis* have been investigated, immune defense mechanisms in the Asian honey bee *Apis cerana* remain unclear. This study examined whether the lncRNA6470/ace-miR-750-y axis modulates the response of *A. cerana* larvae to *A. apis* infection. Stem-loop RT-PCR, Sanger sequencing, dual-luciferase reporter assays, RNA interference, and RT-qPCR were used to characterize ace-miR-750-y, lncRNA6470, *AcPP2A*, host immune genes, and selected *A. apis* genes associated with signal transduction, transcriptional regulation, and RNA metabolism. Larval survival and chalkbrood incidence were also evaluated after ncRNA perturbation. ace-miR-750-y was expressed in larval guts and showed infection-associated expression changes. Computational prediction identified 214 candidate mRNA targets and 143 candidate lncRNA interactors of ace-miR-750-y, from which the lncRNA6470/ace-miR-750-y/*AcPP2A* axis was selected for experimental validation. Modulation of ace-miR-750-y significantly affected *AcPP2A* expression. ace-miR-750-y overexpression increased host immune genes associated with antimicrobial peptide production and peroxidase activity, whereas its inhibition reduced their expression. Modulation of ace-miR-750-y was also associated with altered expression of selected *A. apis* genes and changes in larval survival and chalkbrood incidence. These findings suggest that ace-miR-750-y may play an important regulatory role in the immune response of *A. cerana* worker larvae to *A. apis* infection, and that its interaction with lncRNA6470 may contribute to ceRNA-like regulation during fungal challenge.

## GRAPHICAL ABSTRACT

**Figure:**
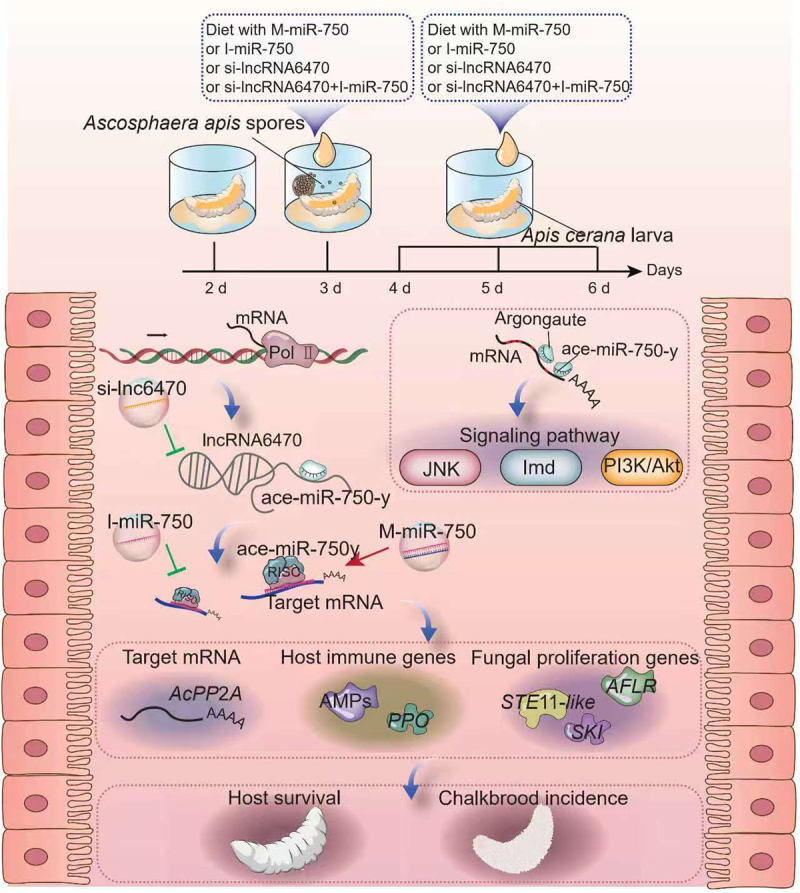

## Introduction

Honey bees provide essential pollination services in agricultural ecosystems and support the production of many pollinator-dependent crops [1,2]. Chalkbrood, caused by the filamentous fungus *Ascosphaera apis*, is a widespread brood disease that kills honey bee larvae and can impair colony renewal and productivity [3]. Because brood loss can ultimately affect colony growth and pollination capacity, understanding honey bee larval diseases has relevance for both apiculture and pollination-dependent agriculture [4]. Therefore, defining the molecular mechanisms that underlie larval defense against *A. apis* is vital for understanding honey bee disease resistance.

Non-coding RNAs, including long non-coding RNAs (lncRNAs) and microRNAs (miRNAs), have emerged as critical regulators of gene expression and immune responses. Among these regulatory mechanisms, lncRNA–miRNA interactions can influence miRNA availability and thereby modulate the expression of miRNA target genes [5,6]. According to the competing endogenous RNA (ceRNA) hypothesis, lncRNAs and other transcripts may regulate mRNA expression by competing for shared miRNA response elements (MREs). Such interactions can fine-tune post-transcriptional gene regulation [7]. This regulatory layer has emerged as a crucial aspect of how insects respond to pathogenic challenges, offering potential pathways for developing novel disease control strategies [8,9]. The ceRNA hypothesis has been extensively validated across diverse organisms, including humans, plants, and animals, and has shed light on the molecular mechanisms of disease pathogenesis and host-pathogen interactions [10,11]. In humans, lncRNA-mediated ceRNA regulation has also been implicated in virus–host interactions. The pseudogene-derived lncRNA PCNAP1 enhances HBV replication by sponging miR-154, thereby increasing *PCNA* expression and promoting HBV cccDNA accumulation [12]. However, experimentally validated lncRNA–miRNA–mRNA modules remain limited in insect–pathogen interaction systems. In the silkworm *Bombyx mori*, Lnc_209997 was reported to suppress BmNPV replication through interaction with miR-275-5p, whereas BmNPV infection downregulated Lnc_209997 and thereby favored viral proliferation [13]. In *Aedes aegypti*, Wolbachia-mediated suppression of aae-lnc-2268 enhances antiviral resistance against dengue virus by modulating the aae-miR-34-3p/MyD88-dependent Toll signaling axis [14]. These examples suggest that lncRNA–miRNA interactions can affect insect immune pathways, but comparable regulatory modules in honey bee antifungal responses remain poorly characterized. Recent transcriptomic studies have highlighted the differential expression of ncRNAs in honey bees infected with fungal pathogens, indicating that ceRNA mechanisms may participate in modulating immune responses during infection [15]

Here, we investigated whether an infection-responsive lncRNA–miRNA–mRNA module contributes to the larval gut response of *Apis cerana* to *A. apis* infection. Previous omics studies of *A. cerana* worker larvae have revealed extensive alterations in mRNA, miRNA, and lncRNA expression in response to *A. apis* invasion [16], providing a basis for investigating ceRNA-mediated regulation. In honey bees, miRNA profiling after *A. apis* challenge has uncovered infection-associated miRNAs whose predicted targets are involved in energy metabolism, cellular and humoral immunity, JAK-STAT signaling, and phagosome-related processes [17]. These findings demonstrate that specific miRNAs and lncRNAs may participate in the modulation of larval antifungal responses. ace-miR-750-y was prioritized since it was abundant in larval gut samples, showed infection-associated expression changes after *A. apis* challenge, and was predicted to target genes associated with cellular or immune-related processes [18]. miR-750 has been identified in several arthropods, including insects such as *B. mori* and *Manduca sexta*. Although early studies mainly described miR-750 in small-RNA catalogs or developmental expression profiles, recent functional studies have linked this miRNA to host-pathogen interactions. In the small brown planthopper *Laodelphax striatellus*, miR-750-3p targets POP7 and suppresses the propagation and transmission of rice black-streaked dwarf virus [19]. In contrast, in the black tiger shrimp *Penaeus monodon*, pmo-miR-750 was upregulated during the late stage of white spot syndrome virus infection and promoted viral propagation by repressing sarcoplasmic calcium-binding protein expression [20]. These findings indicate that miR-750 may participate in pathogen-associated regulation, although its function appears to depend on host species, pathogen type, infection stage, and target-gene context. Whether ace-miR-750-y contributes to antifungal responses in the honey bee larval gut remains unknown.

To investigate the functional relevance of ace-miR-750-y, we predicted its candidate lncRNA interactors and assessed their subcellular localization using computational tools. We then focused on lncRNA6470 and examined whether the lncRNA6470/ace-miR-750-y/*AcPP2A* regulatory axis contributes to the larval immune response during *A. apis* infection. Although high-throughput sequencing has predicted numerous lncRNA-associated ceRNA networks involved in honey bee responses to fungal infection [15], experimental validation of specific lncRNA–miRNA–mRNA modules remains limited. Therefore, this study provides functional evidence for a candidate ncRNA-mediated regulatory axis in the honey bee–*A. apis* interaction and offers new insights into ceRNA-like regulation in insect antifungal immunity.

## Materials and methods

### Fungal activation and spore purification

Based on the established procedure by Chen et al. [21], *A. apis* was cultured on potato dextrose agar (PDA) medium at 33°C for 10 d. Black spore cysts were collected into RNA-free tubes and suspended in DEPC-treated water. The suspension was homogenized to release individual spores and centrifuged to remove impurities. The purified spore suspension was diluted, counted using a hemocytometer, adjusted to the required concentration, and stored at 4°C until use.

### Preparation of larval gut samples

Spore inoculation, artificial rearing, and gut sample preparation of *A. cerana* worker larvae were performed according to the protocol established in our laboratory [22]. Briefly, brood combs were collected from three strong *A. cerana* colonies maintained under the same management conditions in the same apiary at Fujian Agriculture and Forestry University. Two-day-old larvae were selected in the laboratory and individually transferred into 48-well cell culture plates. Each well was preloaded with 50 µL of artificial diet consisting of 63% royal jelly, 30% sterile water, 6% honey, and 1% yeast extract. The 48-well plates were maintained in an incubator at 35 ± 0.5°C and 90% relative humidity. When larvae reached 3-day-old stage, they were randomly assigned to a control group and an *A. apis*-inoculated group. Larvae (*n* = 48) in the control group were fed 50 µL of spore-free artificial diet per larva, whereas larvae (*n* = 48) in the treatment group were inoculated with an equal volume of artificial diet containing *A. apis* spores at a final concentration of 1 × 10^6^ spores/mL. After inoculation, the diet in both groups was replaced every 24 h with fresh spore-free diet, and larvae were reared until 6-day-old. Gut tissues were dissected from 4-, 5-, and 6-day-old larvae under aseptic conditions in a laminar-flow hood. For each treatment at each time point, three biological replicates were prepared, and each biological replicate consisted of pooled guts from three larvae collected into one RNA-free centrifuge tube. The collected gut samples were immediately frozen in liquid nitrogen and stored at −80°C until further analysis. Gut samples from 4-, 5-, and 6-day-old larvae in the control group were designated AcCK1, AcCK2, and AcCK3, whereas the corresponding samples from the *A. apis*-inoculated group were designated AcT1, AcT2, and AcT3, respectively.

### Stem-loop reverse transcription PCR (RT-PCR) and Sanger sequencing

In our previous study, sRNA-seq and bioinformatics were utilized to conduct comprehensive identification and comparative investigation of miRNAs in the guts of *A. cerana* worker larvae following *A. apis* inoculation, identifying 537 conserved and specifically expressed miRNAs [23]. Based on the obtained sequencing data, the top 100 host miRNAs with the highest TPM values were selected, and Venn analysis was then performed to identify the common differentially expressed host miRNAs among the AcCK1 vs AcT1, AcCK2 vs AcT2, and AcCK3 vs AcT3 comparison groups. Special attention was given to the top 10 miRNAs. Given the important role of miR-750 in various processes like immune defense in invertebrates [20,24], ace-miR-750-y was selected for further study. Oligonucleotides for ace-miR-750-y detection, including stem-loop, forward, and universal reverse primers, were designed according to the described method by Guo et al. [25]. Total RNA was extracted from the guts of 6-day-old larvae (*n* = 3) using the Steady Pure Quick RNA Extraction Kit and reverse-transcribed into cDNA with stem-loop primers. The obtained cDNA was then amplified by PCR. After purification, cloning and bacterial transformation, the sample was cultured in LB medium with ampicillin for 12 h. Finally, positive colonies were sequenced by Sangon Biotech using the M13 primers.

### RT-qPCR analysis of ace-miR-750-y expression

Total RNA was isolated from the gut samples of 4-, 5-, and 6-day-old larvae (3 biological replicates per group, each biological replicate included 3 larval guts) and aliquoted into two parallel reverse transcription reactions. Two reverse transcription reactions were performed: one for U6 snRNA and the other for ace-miR-750-y. U6 and ace-miR-750-y were reverse-transcribed using the primer systems specified by the assay protocol. SYBR Green master mix (Yeasen, China) was used for quantitative real-time PCR on a Tianlong Technology thermal cycler, following the manufacturer’s instructions.

### Prediction and annotation of ace-miR-750-y-targeted mRNAs

Potential mRNA targets of ace-miR-750-y were predicted using three computational tools: TargetScan (version 7.0) [26], RNAhybrid (version 2.1.2), combined with svm_light (version 6.01) [27], and miRanda (version 3.3a) [28]. High-confidence targets were designated as those commonly detected by all three tools. Functional interpretation of these candidate genes was subsequently performed via BLAST alignment against the GO (http://geneontology.org/) and KEGG (http://www.genome.jp/kegg/) databases. Data visualization and summary plots were generated using the OmicShares platform (www.omicshare.com). To control the false positive rate, the calculated *p*-values were subjected to False Discovery Rate (FDR) correction. Ultimately, pathways with an FDR-corrected *p* < 0.05 were defined as significantly enriched. Drawing upon KEGG pathway annotations and prior literature [29,30], a subset of targets implicated in pivotal biological processes–namely apoptosis, endocytosis, lysosomal function, melanogenesis, and phagosome activity–as well as key signaling pathways (e.g. Jak-STAT, MAPK, Hippo, FoxO, Wnt, and mTOR) was curated. The integrative regulatory network linking ace-miR-750-y with the selected targets was ultimately reconstructed and visualized in Cytoscape (v3.6.1) [31].

### Dual-luciferase reporter assay

Potential binding sites of ace-miR-750-y in the *AcPP2A* 3’-UTR and lncRNA6470 were predicted using RNAhybrid v2.1.2, following previously described methods by Lan et al. [32] and Yuan et al. [33]. Wild-type fragments containing the predicted binding sites and the corresponding mutant fragments were synthesized by Sangon Biotech (Shanghai, China). These fragments were inserted into the pmirGLO Dual-Luciferase reporter vector (Promega, USA) through the *XhoI* and *SacI* restriction sites.

The recombinant vectors were constructed by ligating the synthesized fragments into linearized pmirGLO vectors with T4 DNA ligase at 16°C overnight. The ligation products were transformed into DH5α competent cells and plated on LB agar plates containing ampicillin. Positive colonies were screened by PCR and further verified by Sanger sequencing. Plasmids from confirmed colonies were extracted using the EasyPure® Plasmid MiniPrep Kit for subsequent transfection assays.

HEK293T cells were maintained at 37°C in a humidified atmosphere with 5 % CO_2_. For the luciferase assay, cells were co-transfected with ace-miR-750-y mimic or mimic negative control, together with wild-type or mutant pmirGLO reporter plasmids, using Hieff Trans^TM^ reagent. For each candidate target, four groups were included: WT+M-NC, WT+M-miR-750-y, MUT+M-NC, and MUT+M-miR-750-y. The same strategy was employed to assess the interaction between ace-miR-750-y and lncRNA6470.

Luciferase activity was detected at 24 h following transfection using the dual-luciferase reporter assay system. Firefly and Renilla luciferase activities were measured sequentially after cell lysis, and relative luciferase activity was calculated as the ratio of firefly to Renilla activity. Three independent biological replicates were performed for each assay.

### Fluorescence in situ hybridization (FISH)

Fluorescence *in situ* hybridization (FISH) was performed to assess the subcellular localization of lncRNA6470 and ace-miR-750-y in the guts of 6-day-old *A. cerana* worker larvae. Cy3-labeled probes targeting lncRNA6470 and FAM-labeled probes targeting ace-miR-750-y were used, and probe information is presented in Supplementary Table S1. Larval guts were fixed in 4% paraformaldehyde for 2 h, dehydrated through a graded ethanol series, cleared in xylene, embedded in paraffin, and sectioned at 4–5 μm thickness. Paraffin sections were baked at 60°C, deparaffinized in xylene, and rehydrated through a graded ethanol series. After washing with PBS, sections were treated with 0.25% HCl for 5–10 min and digested with proteinase K at 10–20 μg/mL for 10–20 min at 37°C. The sections were then pre-hybridized in hybridization buffer and hybridized with the Cy3-labeled lncRNA6470 probe and FAM-labeled ace-miR-750-y probe at 65°C for 48 h in a humidified chamber. After hybridization, sections were washed under stringent conditions, counterstained with DAPI, and mounted with antifade mounting medium. Fluorescence signals were captured using a Nikon ECLIPSE Ts2 fluorescence microscope under identical exposure settings. Cytoplasmic signal overlap was interpreted when the Cy3-labeled lncRNA6470 signal and the FAM-labeled ace-miR-750-y signal overlapped in the merged image within the same cytoplasmic region.

### miRNA overexpression and knockdown

Based on the ace-miR-750-y sequence, ace-miR-750-y inhibitor (I-miR-750), Inhibitor negative control (I-NC), ace-miR-750-y mimic (M-miR-750), and mimic negative control (M-NC) were designed and synthesized by Shanghai GenePharma Co., Ltd. Three-day-old larvae (*n* = 192) were transferred into 48-well culture plates preloaded with artificial diet, inoculated with *A. apis* spores as described above, and randomly assigned to four treatment groups. For miRNA overexpression, larvae (*n* = 48) were each fed 50 µL of diet containing M-miR-750 or M-NC at a final concentration of 40 pmol/g diet. For miRNA inhibition, larvae were fed 50 µL of diet containing I-miR-750 or I-NC at a final concentration of 80 pmol/g diet. Each treatment included three biological replicates. Fresh diet containing the corresponding mimic, inhibitor, or negative control was supplied every 24 h for three consecutive feedings.

### Quantification of *AcPP2A*, host immune genes, and selected *A. apis* genes

RNA extracted from gut samples from the AcCK1, AcCK2, AcCK3, AcT1, AcT2, and AcT3 groups was reverse-transcribed into cDNA using random primers and Oligo(dT)_18_. The expression levels of *AcPP2A* (GenBank accession number: XM_062085879.1), as well as the immune-related genes *Dorsal* (GenBank accession number: XM_017053960.2), *Defensin* (GenBank accession number: XM_017050425.2), *Hymenoptaecin* (GenBank accession number: XM_017049926.1), and *PPO* (GenBank accession number: NM_001011627.1), were quantified by RT-qPCR. The *actin* gene (GenBank accession number: XM_017066822.2) served as a control for normalization.

The expression levels of three selected *A. apis* genes were also examined in larval gut samples, including *STE11-like* (GenBank accession number: EF156415.1), *AFLR* (GenBank accession number: KZZ91552.1), and *SKI* (GenBank accession number: KZZ98039.1), related to signal transduction, transcriptional regulation, and RNA metabolism, respectively. The *A. apis* 5.8*S rRNA* gene (GenBank accession number: U68313.1) was used as the reference gene for fungal gene expression analysis. The RT-qPCR reaction mixture and cycling conditions were the same as those described above.

### Screening of candidate ace-miR-750-y-interacting lncRNAs

Candidate upstream lncRNAs interacting with ace-miR-750-y were predicted using previously generated high-quality transcriptome data. LncRNAs with a predicted binding free energy of less than −21 kcal/mol were selected for further analysis. The subcellular localization of the identified lncRNAs was predicted using the iLoc-lncRNA online tool, and candidate lncRNAs predicted to localize to the cytoplasm were selected for further analysis.

### Determination of lncRNA expression profile

RT-qPCR primers for lncRNA6470 were designed using Primer Premier 6 (Premier Biosoft, Palo Alto, CA, USA). Extracted RNA was divided into two aliquots, with one portion was reverse transcribed using Random primers to generate cDNA templates for lncRNA6470 RT-qPCR, one portion reverse transcribed using Random and Oligo (dT)_18_ primers to generate cDNA templates for *actin* as the internal reference.

### Measurement of lncRNA6470 expression after ace-miR-750-y overexpression and inhibition

After knockdown or overexpression of ace-miR-750-y in the *A. cerana* larval gut, total RNA was extracted from guts. Reverse transcription and RT-qPCR were then performed to determine the relative expression level of lncRNA6470 using the protocol described above.

### RNAi of lncRNA

siRNA targeting lncRNA6470 (si-lncRNA6470) and a non-targeting control (si-scramble) were designed and synthesized. Following the above-described feeding method used for the preparation of *A. cerana* gut samples, 3-day-old larvae were infected by *A. apis*, and fed with si-lncRNA6470 or si-scramble (2 μg/larva) for three consecutive days, with daily supplementation. Larvae were preserved as described.

RNA from gut tissue was isolated and used for RT-qPCR analysis of U6 snRNA and ace-miR-750-y according to the protocol described above. RNA from 4–6-day-old larvae was also reverse transcribed using Random primers and Oligo(dT)18, and real-time quantitative PCR was employed to assess *AcPP2A* and immune gene expression levels (*Dorsal*, *Defensin*, *Hymenoptaecin*, *PPO*), and pathogen-related genes (*STE*11-*like*, *AFLR*, *SKI*), following the aforementioned procedure.

### Host survival rate and chalkbrood incidence

According to the procedure described above, 3-day-old larvae were inoculated with *A. apis* spores (3 biological replicates; *n* = 24). Starting from the day of inoculation (0 dpi), the larvae were fed daily with either si-lncRNA6470, si-scramble, M-miR-750, I-miR-750, M-NC, or I-NC until they reached 10 days of age. Survival rates across the treatment and control groups, as well as the incidence of chalkbrood disease in the host, were monitored and statistically analyzed on a daily basis.

### Rescue assay via simultaneous lncRNA6470 silencing and ace-miR-750-y inhibition

**Figure 1.**
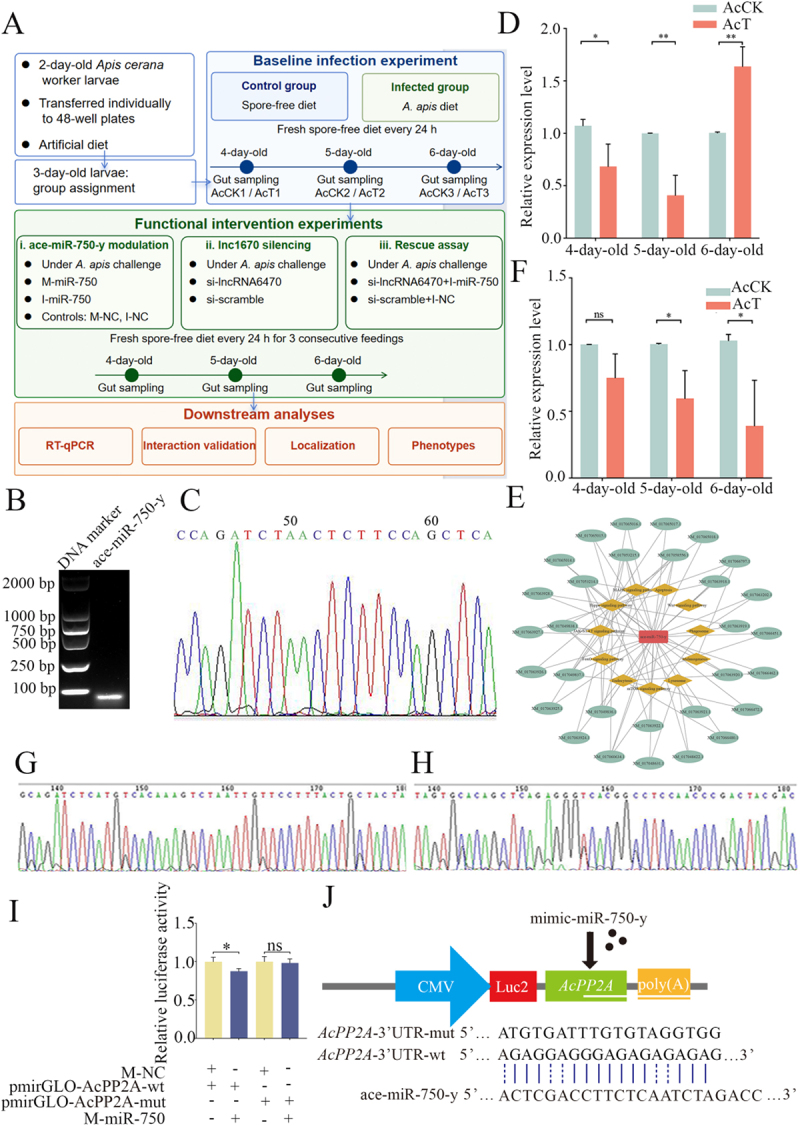
Molecular verification, target annotation, and binding relationship validation of ace-miR-750-y. (A) Experimental workflow used to investigate the lncRNA6470/ace-miR-750-y/*AcPP2A* regulatory axis in *A. cerana* larvae during *A. apis* infection. (B) Agarose gel electrophoresis showing the amplification product from ace-miR-750-y. (C) Sanger sequencing results of the amplified product. (D) ace-miR-750-y expression levels in *A. cerana* larval guts following *A. apis* inoculation. (E) Regulatory network between ace-miR-750-y and target mRNAs. (F) Relative expression level of *AcPP2A* in the guts of *A. apis*-inoculated larvae. (G-H) Sanger sequencing chromatograms for recombinant plasmids pmirGLO-AcPP2A-wt and pmirGLO-AcPP2A-mut. (I) Dual-luciferase reporter assay validating the interaction between ace-miR-750-y and *AcPP2A*. (J) Schematic diagram of the predicted ace-miR-750-y binding site in the *AcPP2A* 3’-UTR and the corresponding mutated sequence used for the dual-luciferase reporter assay. Data are shown as mean ± SD (*n* = 3). ns, not significant; *p < 0.05; **p < 0.01; ***p < 0.001; ****p < 0.0001. WT, wild type; MUT, mutant; NC, negative control.

For the rescue assay, 3-day-old larvae (*n* = 72) were randomly assigned to treatment and control groups. In the treatment group (36 larvae in total), each larva was initially fed 50 μL of artificial diet containing *A. apis* spores (5 × 10^4^), 2 μg of si-lncRNA6470, and 2 μg of I-miR-750. In the control group (36 larvae in total), each larva received the same volume of diet containing *A. apis* spores, siRNA-scramble, and I-NC. After the initial diet was consumed, larvae were supplied every 24 h for three consecutive feedings with fresh spore-free diet containing the corresponding siRNA and inhibitor treatments. RNA from the larval gut samples in each group was isolated with a RNA extraction kit. Subsequent analysis of the relative expression of *AcPP2A*, immune response genes, and selected *A. apis* genes associated with signaling, transcriptional regulation, and RNA metabolism was performed following the procedures described above. The experimental workflow used to investigate the lncRNA6470/ace-miR-750-y/*AcPP2A* regulatory axis in *A. cerana* larvae during *A. apis* infection is shown in Figure 1(A).

### Statistical analysis

For RT-qPCR, each biological replicate consisted of pooled guts from three larvae, and three independent biological replicates were analyzed per treatment and time point. For survival and incidence assays, each treatment included three replicate plates with 24 larvae per replicate.

Statistical analyses were performed using IBM SPSS Statistics 27 (IBM Corp., Armonk, NY, USA) and GraphPad Prism 8 (GraphPad Software, San Diego, CA, USA). Relative expression levels were calculated using the 2^−ΔΔCt^ method [34], and expression changes were presented as log_2_ fold change. Data were assumed to be normally distributed based on the nature of the biological experiments. For RT-qPCR, and dual-luciferase reporter assays, statistical significance for multiple comparisons was determined using multiple *t*-tests followed by the Holm-Sidak correction. Survival data were analyzed using Kaplan-Meier estimates and compared using the log-rank test. Chalkbrood incidence was analyzed using a generalized linear model with a binomial error distribution. Data are presented as mean ± SD unless otherwise stated.

## Results

### Molecular confirmation and target prediction of ace-miR-750-y

Agarose gel electrophoresis showed successful amplification of a fragment of approximately 100 bp, verifying the expression of ace-miR-750-y in the *A. cerana* worker larval guts (Figure 1(B)). Sanger sequencing confirmed that the amplified fragment was identical to the predicted sequence of ace-miR-750-y (Figure 1(C)). As shown in Figure 1(D), ace-miR-750-y expression was significantly downregulated in the 4- and 5-day-old larval guts following *A. apis* inoculation compared with the corresponding controls (*p* < 0.05), but was significantly upregulated in the 6-day-old larval gut (*p* < 0.01).

A predicted ace-miR-750-y-centered regulatory network was constructed based on 214 candidate mRNA targets (Figure 1(E)). Functional annotation assigned these targets to 25 GO terms in three categories: biological process, cellular component, and molecular function. These GO terms were mainly associated with cellular processes, cellular components, binding, catalytic activity, and signal transduction. In addition, 124 KEGG pathways annotated by these targets were identified, including those relevant to cellular processes, environmental information processing, genetic information processing, and metabolism. Notably, several targets were associated with key processes: eight with endocytosis, two with lysosome, and one each with apoptosis, melanogenesis, and phagosome. Pathway analysis further indicated enrichment of target mRNAs in Hippo (17), Jak-STAT (6), MAPK (6), FoxO (6), mTOR (3), and Wnt (1) signaling pathways (Figure 1(E)). The effect of *A. apis* infection on *AcPP2A* expression was further examined. *AcPP2A* expression was reduced in the 4-day-old larval guts, although the difference was not statistically significant, and was significantly downregulated in the guts of 5- and 6-day-old larvae following *A. apis* inoculation (Figure 1(F)).

### Validation of the interaction between ace-miR-750-y and *AcPP2A*

Sanger sequencing confirmed the successful construction of recombinant plasmids containing the wild-type and mutant target sequences, respectively named pmirGLO-AcPP2A-wt and pmirGLO-AcPP2A-mut (Figure 1(G,H)). Dual-luciferase assays demonstrated a sequence-specific interaction between ace-miR-750-y and the binding site in the *AcPP2A* 3’-UTR. Compared with the pmirGLO-AcPP2A-mut plasmids, relative luciferase activity was significantly decreased (*p* < 0.001) after co-transfection of M-miR-750 and pmirGLO-AcPP2A-wt plasmids. Co-transfection of M-miR-750 with pmirGLO-AcPP2A-mut did not significantly alter relative luciferase activity (*p* > 0.05) (Figure 1(I)).

### Modulation of ace-miR-750-y affects *AcPP2A* expression in *A. apis*-challenged larval guts

**Figure 2.**
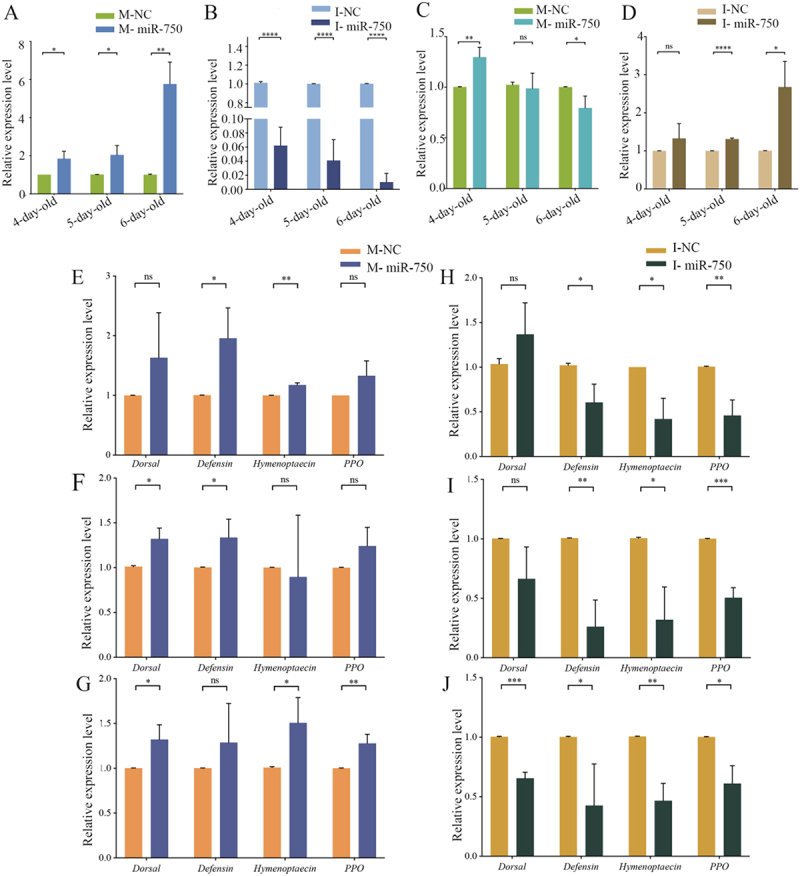
Effect of ace-miR-750-y overexpression and inhibition on *AcPP2A* expression and immune response regulation in *A. cerana* larvae. (A-B) Relative expression levels of ace-miR-750-y in the guts of *A. cerana* larvae after feeding with M-miR-750 and I-miR-750. (C-D) Relative expression levels of *AcPP2A* in the larval guts following ace-miR-750-y overexpression and inhibition. (E-J) Immune gene expression profiles (*Dorsal, Defensin*, *Hymenoptaecin, PPO*) in larval guts after feeding with M-miR-750 vs M-NC (E-G) or I-miR-750 vs I-NC (H-J). Mimic-miR-750, ace-miR-750-y mimic, mimic-NC, mimic negative control; inhibitor-miR-750, ace-miR-750-y inhibitor; inhibitor-NC, inhibitor negative control.

RT-qPCR analysis revealed distinct expression patterns following experimental modulation of ace-miR-750-y. Compared to the M-NC group, ace-miR-750-y expression was significantly upregulated in the larval guts of 4-, 5-, and 6-day-old individuals in the M-miR-750 group (Figure 2(A)). Conversely, feeding with I-miR-750 resulted in a marked downregulation (*p* < 0.0001) of ace-miR-750-y in *A. apis*-inoculated larvae across all three ages (Figure 2(B)). Overexpression of ace-miR-750-y differentially influenced *AcPP2A* expression: a significant upregulation (*p* < 0.01) was observed in the 4-day-old larval guts, a non-significant increase (*p* > 0.05) in the 5-day-old larval gut, and a significant downregulation (*p* < 0.05) in the 6-day-old larval guts, following *A. apis* inoculation (Figure 2(C)). In contrast, I-miR-750 feeding led to a non-significant *AcPP2A* upregulation (*p* > 0.05) in the 4-day-old larval guts, and significant upregulation (*p* < 0.05) in the guts of 5- and 6-day-old larvae inoculated with *A. apis* (Figure 2(D)).

### Effect of ace-miR-750-y modulation on host immune gene expression in *A. apis*-infected larval guts

Overexpression of ace-miR-750-y increased the expression of host immune genes associated with antimicrobial peptides and peroxidase activity (Figure 2(E-G)). In comparison with the M-NC group, the M-miR-750 treatment significantly increased *Defensin* and *Hymenoptaecin* expression (*p* < 0.05) in the 4-day-old larval gut. In the same group, the transcript levels of *Dorsal* and *PPO* were also increased, as illustrated in Figure 2(E). In the 5-day-old larval gut, the expression levels of *Dorsal* and *Defensin* were significantly increased (*p* < 0.05) (Figure 2(F)). As shown in Figure 2(G), the expression levels of *Dorsal*, *Hymenoptaecin*, and *PPO* were significantly upregulated in the 6-day-old larval guts (*p* < 0.05). Following ace-miR-750-y knockdown, the expression levels of *Defensin* and *Hymenoptaecin* were significantly reduced (*p* < 0.05) in the guts of 4-, 5-, and 6-day-old larvae, while the *PPO* expression was significantly reduced in all stages tested (*p* < 0.01 for 4-day-old, *p* < 0.001 for 5-day-old, *p* < 0.05 for 6-day-old). The *Dorsal* expression was significantly down-regulated (*p* < 0.001) only in the 6-day-old larval guts (Figure 2(H-J)).

### Effects of ace-miR-750-y modulation on selected *A. apis* genes related to signaling, transcriptional regulation, and RNA metabolism

**Figure 3.**
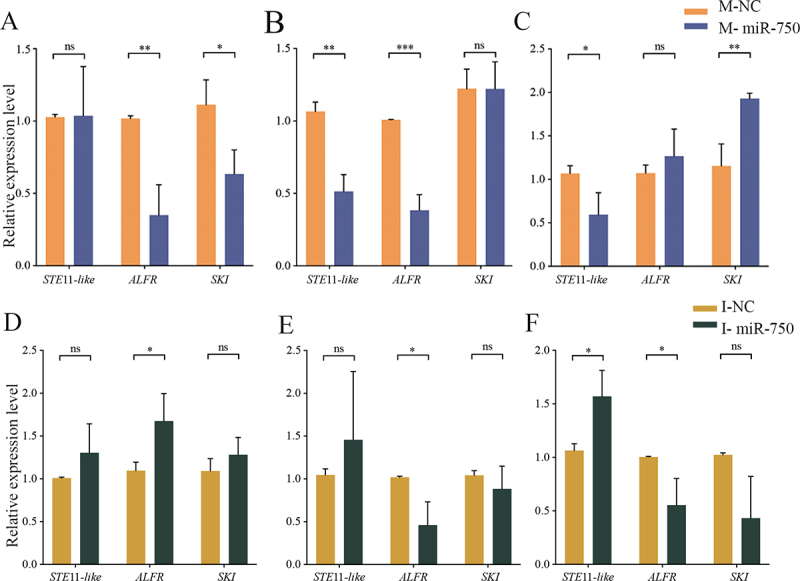
Effects of ace-miR-750-y modulation on the expression of selected *A. apis* genes associated with signaling, transcriptional regulation, and RNA metabolism during larval infection. (A-C) Relative expression levels of *STE*11-*like, AFLR*, and *SKI* after feeding with M-miR-750 and M-NC. (D-F) Relative expression levels of *STE*11-*like, AFLR*, and *SKI* after feeding with I-miR-750 and I-NC.

Modulation of ace-miR-750-y expression differentially influenced *A. apis* genes across larval development. Following ace-miR-750-y overexpression, the significantly downregulation of *AFLR* (*p* < 0.01) and *SKI* (*p* < 0.05) was observed in the 4-day-old larval guts, while *STE*11-*like* remained unchanged (Figure 3(A)). In 5-day-old larval guts, the *STE*11*-like* (*p* < 0.01) and *AFLR* (*p* < 0.001) were significantly suppressed, with *SKI* unaffected (Figure 3(B)). In 6-day-old larval guts, the *STE*11*-like* was significantly downregulated (*p* < 0.05) while *SKI* was significantly elevated (*p* < 0.01) (Figure 3(C)).

Inhibition of miR-750 similarly gave rise to stage-specific expression alterations. In 4-day-old larval guts, significant upregulation (*p* < 0.05) of *AFLR* was detected in the larval guts (Figure 3(D)). The *AFLR* expression was decreased (*p* < 0.05) in the 5-day-old larval guts (Figure 3(E)). As shown in Figure 3(F), the *STE*11*-like* expression was significantly induced (*p* < 0.05) whereas the *AFLR* expression was reduced (*p* < 0.05) in 6-day-old larval guts.

### Prediction and screening of ace-miR-750-y-interacting lncRNAs

**Figure 4.**
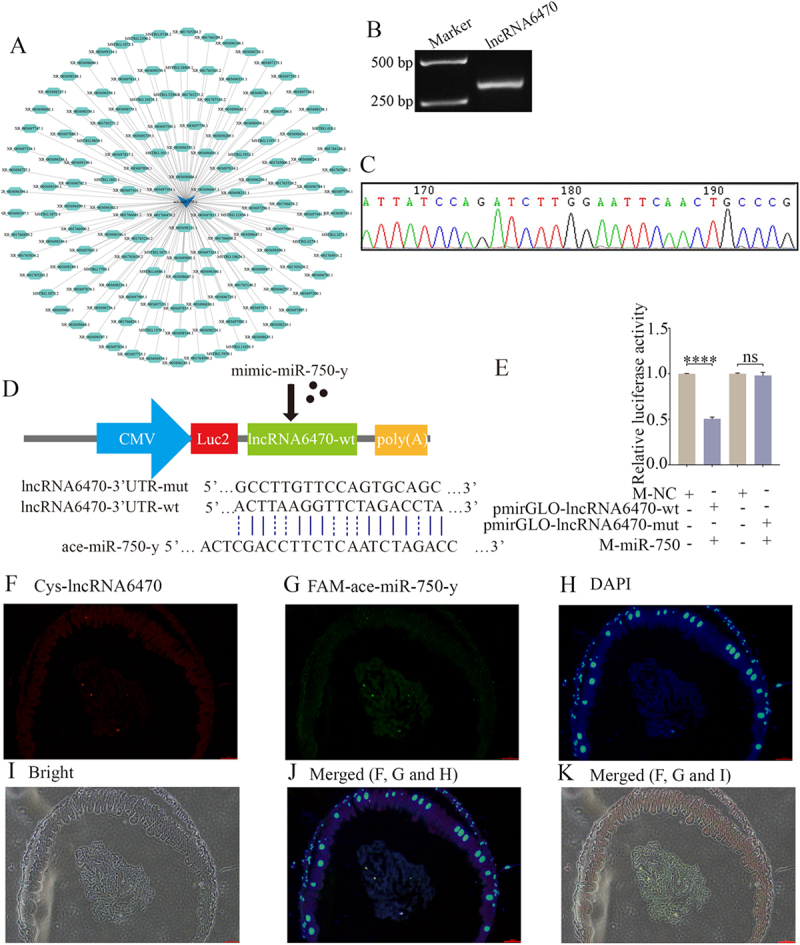
Predicted ace-miR-750-y/lncRNA regulatory network, validation of the interaction between ace-miR-750-y and lncRNA6470, and overlapping cytoplasmic signals in larval guts. (A) ace-miR-750-y and target lncRNA regulatory interplay. (B) PCR amplification of the predicted binding site between ace-miR-750-y and lncRNA6470. (C) Sanger sequencing of the amplified fragment. (D) Schematic representation of the dual-luciferase reporter assay vector design, including the predicted binding sequences of ace-miR-750-y and wild-type (wt) or mutated (mut) lncRNA6470 fragments containing the predicted ace-miR-750-y binding site. (E) Dual-luciferase reporter assay validating the interaction between ace-miR-750-y and lncRNA6470, with the wild-type (wt) and mutant (mut) target sequences cloned into pmirGLO vectors. (F-H) Fluorescence *in situ* hybridization (FISH) showing Cy3-labeled lncRNA6470 (red), FAM-labeled ace-miR-750-y (green), and DAPI-stained nuclei (blue) in larval gut sections. (I) Bright-field image of the larval gut section. (J-K) Merged images of FISH results. Scale bars = 50 μm.

A total of 143 lncRNAs were predicted as potential ace-miR-750-y-interacting candidates. This lncRNA-miRNA network was used to screen cytoplasmic lncRNAs for subsequent experimental validation (Figure 4(A)). Based on iLoc-lncRNA prediction, three of these target lncRNAs might be localized in the cytoplasm. Further analysis showed that the expression level of XR_001766470.2 (designated as lncRNA6470) had a potential negative correlation with that of ace-miR-750-y.

### Validation of the interaction between lncRNA6470 and ace-miR-750-y in *A. apis*-infected larval guts

PCR amplification and Sanger sequencing confirmed the successful construction of the pmirGLO-lncRNA6470-miR-750-wt recombinant plasmid (Figure 4(B,C)). Dual-luciferase reporter assays then supported a sequence-dependent interaction between ace-miR-750-y and lncRNA6470 (Figure 4(D,E)). Dual-luciferase assays showed that co-transfection with M-miR-750 and the wild-type reporter plasmid significantly suppressed luciferase activity (*p* < 0.0001), whereas no significant change (*p* > 0.05) was observed with the mutant plasmid (Figure 4(E)). Furthermore, fluorescence *in situ* hybridization (FISH) showed overlapping cytoplasmic signals of lncRNA6470 and ace-miR-750-y, supporting their spatial proximity in larval gut cells (Figure 4(F–K)).

**Figure 5.**
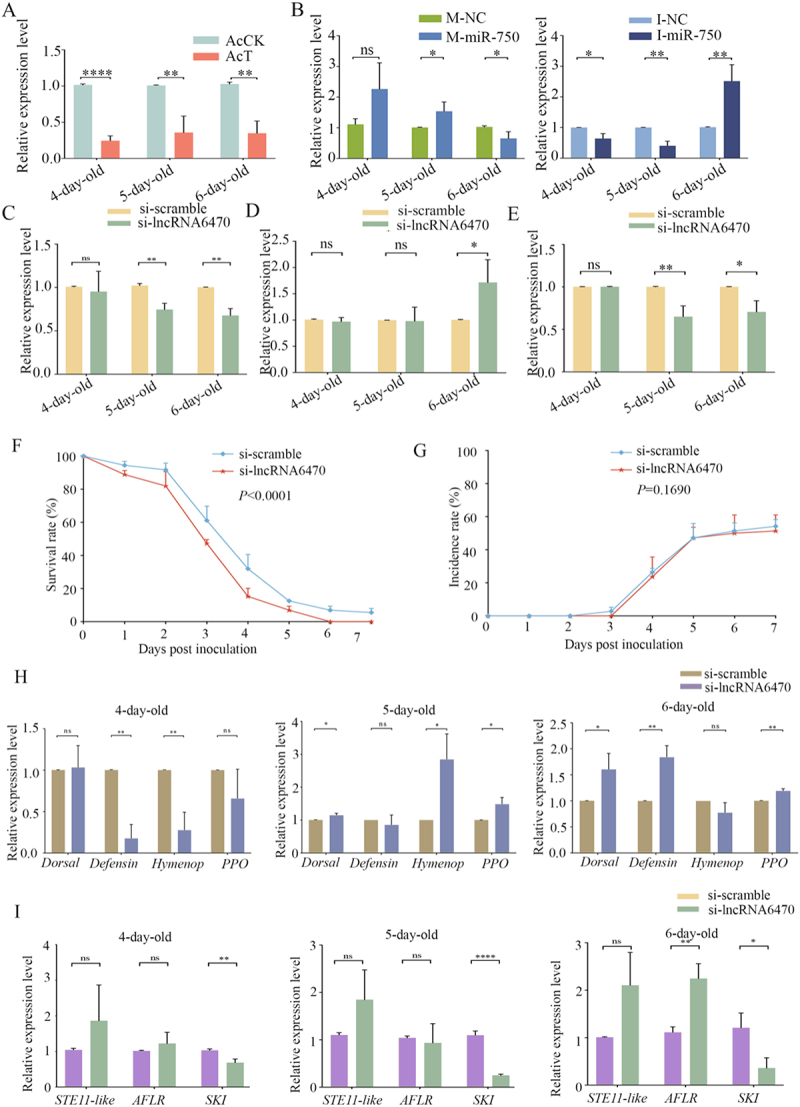
Expression pattern of lncRNA6470 in *A. cerana* larval guts under different treatments and the effects of lncRNA6470 silencing on ceRNAs and host immunity. (A) Relative expression levels of lncRNA6470 in larvae infected with *A. apis*. (B) Expression changes of lncRNA6470 following overexpression and knockdown of ace-miR-750-y. (C) RNAi efficiency of lncRNA6470 silencing in the guts of *A. cerana* worker larvae. (D-E) Relative expression levels of ace-miR-750-y (D) and *AcPP2A* (E) after silencing lncRNA6470. (F) Host survsival rate following lncRNA6470 knockdown. (G) Chalkbrood incidence in larvae after lncRNA6470 silencing. (H) Relative expression levels of host immune-related genes following lncRNA6470 knockdown. (I) Relative expression levels of selected *A. apis* genes associated with signaling, transcriptional regulation, and RNA metabolism after silencing lncRNA6470. Survival data were analyzed using Kaplan-Meier estimates and compared using the log-rank test. Chalkbrood incidence was analyzed as proportion data using logistic regression or a generalized linear model with binomial error distribution.

Expression analysis indicated that *A. apis* infection significantly downregulated lncRNA6470 (*p* < 0.01) across 4-, 5-, and 6-day-old larval guts (Figure 5(A)). Overexpression of ace-miR-750-y resulted in stage-specific lncRNA6470 expression: a non-significant increase (*p* > 0.05) in 4-day-old larval guts, significant upregulation (*p* < 0.05) in 5-day-old larval guts, and significant downregulation (*p* < 0.05) in 6-day-old larval guts compared to M-NC (Figure 5(B)). Conversely, inhibition of ace-miR-750-y significantly suppressed lncRNA6470 expression in 4- and 5-day-old larvae (*p* < 0.05) but induced its expression in 6-day-old larval guts (*p* < 0.01) relative to I-NC (Figure 5(B)).

### LncRNA6470 silencing alters ace-miR-750-y and *AcPP2A* expression

After lncRNA6470 silencing, lncRNA6470 expression showed a non-significant decrease (*p* > 0.05) in 4-day-old larval guts. Its expression was significantly reduced in the guts of 5- and 6-day-old larvae (*p* < 0.01; Figure 5(C)). This silencing induced a significant upregulation of ace-miR-750-y (*p* < 0.05) specifically in 6-day-old larval guts, with no significant changes at earlier ages (Figure 5(D)). Concurrently, the expression level of *AcPP2A* was significantly downregulated (*p* < 0.05) in both 5- and 6-day-old larval guts post-silencing (Figure 5(E)).

### Effects of lncRNA6470 silencing on larval survival, host immune gene expression, and selected *A. apis* transcript levels

Interference with lncRNA6470 resulted in decreased survival rate of larvae inoculated with *A. apis*. Following inoculation, the survival rates in both the si-scramble and si-lncRNA6470 groups progressively declined over time. From 0 to 2 dpi, larval survival decreased gradually in both groups. A sharp decline occurred between 2 and 4 dpi, with survival rates in the si-lncRNA6470 group dropping to 81.94%, 47.22%, and 15.28%, compared to 91.67%, 61.11%, and 31.94% in the si-scramble group. Between 4 and 6 dpi, the decline continued more slowly. By 7 dpi, the survival rate in the si-lncRNA6470 group had dropped to 0%, whereas that in the si-scramble group was 6.95%. Following lncRNA6470 knockdown, larval survival was significantly reduced relative to the si-scramble group (*p* < 0.0001) (Figure 5(F)).

lncRNA6470 knockdown did not significantly alter chalkbrood incidence in *A. apis*-infected larvae. As shown in Figure 5(G), the incidence rate of infection in both the si-scramble and si-lncRNA6470 groups increased progressively over time. During the initial 0–3 dpi, morbidity remained low in both groups. A marked increase was observed between 3 and 5 dpi, followed by a slower rise from 5 to 7 dpi. By 7 dpi, the incidence rates reached 54.17% in the si-scramble group and 51.39% in the si-lncRNA6470 group. No significant difference in morbidity was observed between the two groups (*p* > 0.05) (Figure 5(G)).

Analysis of immune-related gene expression following lncRNA6470 knockdown revealed distinct temporal patterns. *Dorsal* expression was significantly up-regulated at 5- and 6-day-old (*p* < 0.05), though unchanged in 4-day-old larval guts (*p* > 0.05). *Defensin* was significantly down-regulated in 4-day-old larval guts (*p* < 0.01), unchanged in 5-day-old larval guts (*p* > 0.05), and markedly up-regulated in 6-day-old larval guts (*p* < 0.01). *Hymenoptaecin* expression was strongly suppressed in 4-day-old larval guts (*p* < 0.01), elevated in 5-day-old larval guts (*p* < 0.05), and returned to baseline levels by 6-day-old (*p* > 0.05). *PPO* showed no significant change in 4-day-old larval guts (*p* > 0.05) but was significantly up-regulated at both 5-day-old (*p* < 0.05) and 6-day-old (*p* < 0.01) (Figure 5(H)).

Transcriptional analysis of pathogen genes following lncRNA6470 knockdown revealed distinct expression dynamics. The *STE*11-*like* expression did not change significantly at any time point after lncRNA6470 silencing (*p* > 0.05). In contrast, the *AFLR* expression was unchanged in the guts of 4- and 5-day-old larvae (*p* > 0.05) but was significantly upregulated in 6-day-old larval guts (*p* < 0.01), suggesting a delayed regulatory effect. Conversely, the *SKI* expression was consistently and significantly suppressed across all developmental stages, with strong downregulation in 4-day-old larval guts (*p* < 0.01), 5-day-old (*p* < 0.0001), and 6-day-old (*p* < 0.05) post-interference (Figure 5(I)).

### Ace-miR-750-y inhibition partially rescues *AcPP2A* expression and larval survival after lncRNA6470 silencing

**Figure 6.**
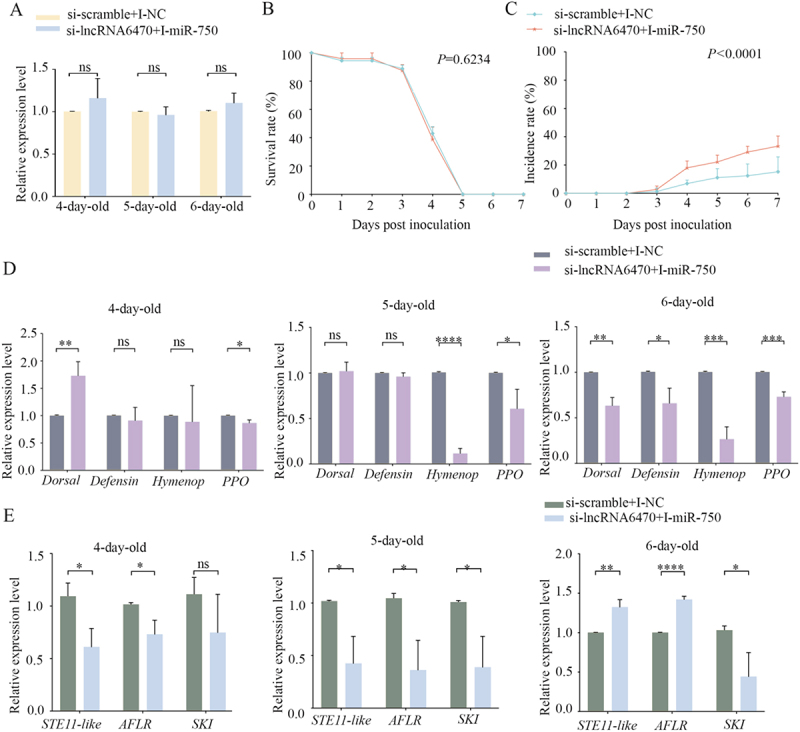
Rescue assay by simultaneous lncRNA6470 silencing and ace-miR-750-y inhibition during *A. apis* infection. (A) Relative expression level of *AcPP2A* after simultaneous lncRNA6470 silencing and ace-miR-750-y inhibition. (B) Host survival rate after simultaneous lncRNA6470 silencing and ace-miR-750-y inhibition. (C) Chalkbrood incidence in larvae after lncRNA6470 silencing and ace-miR-750-y inhibition. (D) The relative expression levels of host immune-related genes after simultaneous lncRNA6470 silencing and ace-miR-750-y inhibition. (E) Relative expression levels of selected *A. apis* genes associated with signaling, transcriptional regulation, and RNA metabolism after simultaneous lncRNA6470 silencing and ace-miR-750-y inhibition.

Simultaneous lncRNA6470 silencing and ace-miR-750-y inhibition did not significantly alter *AcPP2A* expression in larval guts across all time points (*p* > 0.05; Figure 6(A)), nor did it affect larval survival following *A. apis* inoculation, with both the si-scramble + I-NC and si-lncRNA6470 + I-miR-750 groups showing a similar gradual decline in survival (*p* > 0.05; Figure 6(B)). In contrast, co-silencingcombined treatment significantly increased the chalkbrood incidence of larvae: while infection rates remained low until 3 dpi in both groups, they rose sharply thereafter, reaching 84.72% in the si-lncRNA6470 + I-miR-750 group vs 61.11% in controls by 7 dpi (*p* < 0.0001; Figure 6(C)).

In addition, the expression of host immune genes were changed by dual knockdown. In 4-day-old larval guts, *Dorsal* was up-regulated (*p* < 0.01) and *PPO* down-regulated (*p* < 0.05), while *Defensin* and *Hymenoptaecin* remained unchanged. In 5-day-old larval guts, *Hymenoptaecin* and *PPO* were significantly suppressed (*p* < 0.0001 and *p* < 0.05). In 6-day-old larval guts, all four genes were markedly down-regulated: *Dorsal* (*p* < 0.01), *Defensin* (*p* < 0.05), *Hymenoptaecin* (*p* < 0.001), and *PPO* (*p* < 0.001) (Figure 6(D)).

Three selected fungal genes (*STE*11-*like*, *AFLR*, and *SKI*) were analyzed following dual knockdown. in 4-day-old larval guts, both *STE*11-*like* and *AFLR* were significantly down-regulated (*p* < 0.05), whereas *SKI* remained unchanged (*p* > 0.05). In 5-day-old larval guts, all three genes were significantly suppressed (*p* < 0.05). In 6-day-old larval guts, the *STE11-like* and *AFLR* expression was significantly increased (*p* < 0.01 and *p* < 0.0001, respectively), whereas the *SKI* expression was significantly decreased (*p* < 0.05) (Figure 6(E)).

## Discussion

### The expression dynamics and potential regulatory roles of ace-miR-750-y in larval defense against *A. apis* infection

ace-miR-750-y showed a stage-dependent response to *A. apis* infection in the larval gut. Stem-loop RT-PCR and Sanger sequencing confirmed the presence of ace-miR-750-y in *A. cerana* larval guts (Figure 1(B,C)), and RT-qPCR further revealed its infection-associated expression pattern. Following *A. apis* inoculation, ace-miR-750-y was downregulated in the 4- and 5-day-old larval guts but upregulated in the 6-day-old larval gut, indicating a stage-dependent response to fungal infection (Figure 1(D)). This stage-dependent pattern suggests that ace-miR-750-y may participate in the temporal regulation of the larval gut responding to fungal infection. Previous studies in other arthropods have linked miR-750 family members to pathogen-associated regulatory processes [[19,20,35,36]. In shrimp, miR-750 is upregulated during late stage of white spot syndrome virus infection and promotes viral replication by affecting apoptosis-related regulation [20]. In contrast, miR-750-3p in *L. striatellus* suppresses the replication of rice black-streaked dwarf virus by targeting *LsPOP7*, with mimics reducing viral accumulation and inhibitors producing the opposite effect [19]. These studies suggest that miR-750 family members can participate in host–pathogen regulatory networks, although their effects differ among host species and pathogen types.

Target prediction identified a set of ace-miR-750-y-targeted genes with potential involvement in cellular and immune-related processes (Figure 1(E)). Some of these targets were associated with endocytosis, lysosomal function, apoptosis, melanogenesis, and phagosome-related processes. In the larval gut, such processes are likely to interact with conserved immune pathways that regulate antimicrobial peptide production and epithelial defense. Other potential targets were annotated to several vital signaling pathways such as Hippo, JAK-STAT, MAPK, FoxO, mTOR, and Wnt (Figure 1(E)). These data suggest that ace-miR-750-y may contribute to the regulation of immune-related processes by post-transcriptionally modulating its target genes.

### Ace-miR-750-y interacts with *AcPP2A* and regulates its expression in a stage-dependent manner

PP2A is a conserved serine/threonine phosphatase engaged in signal transduction, apoptosis, proliferation, and metabolism [37,38]. In insects, altered *PP*2*A* expression has also been associated with pathogen infection, as reported in the BmNPV–silkworm system [39]. Here, the dual-luciferase reporter assay provided evidence that ace-miR-750-y can directly interact with the binding site in the *AcPP2A* 3’-UTR (Figure 1(I)). The expression level of *AcPP2A* was significantly reduced in the 5- and 6-day-old larval guts following *A. apis* inoculation (Figure 1(F)), indicating that *AcPP2A* was responsive to fungal infection. Notably, *AcPP2A* and ace-miR-750-y showed an inverse expression pattern in 6-day-old infected larvae (Figure 1(D,F)), suggesting a potential negative regulatory relationship between ace-miR-750-y and AcPP2A.

However, the *in vivo* response of *AcPP2A* to ace-miR-750-y modulation was not fully consistent with a simple linear model of miRNA-mediated repression. In particular, ace-miR-750-y overexpression in the larval guts increased the *AcPP2A* expression in 4-day-old larval guts, had no significant effect in 5-day-old larval guts, and reduced its expression in 6-day-old larval guts (Figure 2(C)). This dynamic pattern implies that during the early stage of infection, the acute mRNA disruption induced by the miRNA mimic may trigger a robust transcriptional compensation response–a phenomenon recently documented in insect models [55]. Furthermore, similar observations in *Drosophila* cells indicate that miRNA abundance and target repression are not always linearly correlated [40]. Therefore, the stage-specific response of *AcPP2A* in the present study does not contradict the direct binding between ace-miR-750-y and the *AcPP2A* 3’-UTR. Instead, it suggests that ace-miR-750-y-mediated regulation of *AcPP2A* is modulated by the developmental stage and infection status of the larval gut. Collectively, these results support a direct ace-miR-750-y–*AcPP2A* interaction and reveal that ace-miR-750-y regulates *AcPP2A* expression in a stage-dependent manner during *A. apis* infection (Figure 1(I), Figure 2(C,D)).

### Ace-miR-750-y affects host immune gene expression and *A. apis* genes associated with fungal biology

In addition to directly regulating target gene expression through sequence-specific binding, miRNAs can also influence non-target genes indirectly [41,42]. *Defensin*, *Hymenoptaecin*, and *PPO* are important components of insect humoral immunity and melanization-associated defense [43–46]. In this work, the coordinated changes in AMP-related genes and *PPO* after ace-miR-750-y overexpression or inhibition indicate that this miRNA is associated with immune-gene regulation in infected larval guts (Figure 2(E-J)). These findings suggest that ace-miR-750-y may contribute to the activation or maintenance of immune responses in host guts. Since these immune genes were not validated as direct targets of ace-miR-750-y, their altered expression is likely to reflect indirect regulation through *AcPP2A*-related signaling or other infection-responsive pathways.

STE11-like kinases mediate MAPK signaling for fungal development and stress responses [47]. *AFLR* activates toxin biosynthetic gene clusters [48]. The SKI complex regulates RNA surveillance to maintain fungal RNA quality and antiviral defense [49]. Here, modulation of ace-miR-750-y also altered the expression of selected *A. apis* transcripts associated with signal transduction, transcriptional regulation, and RNA metabolism (Figure 3(A-F)). These changes might reflect stage-dependent effects of ace-miR-750-y on the host intestinal environment or immune pressure rather than direct regulation of fungal transcripts.

### LncRNA6470 interacts with ace-miR-750-y and is associated with its negative regulation

Previous work in *A. cerana* has suggested that infection-responsive lncRNA (lncRNA13164) may participate in miRNA-associated regulatory networks during *A. apis* infection [50]. Subcellular localization of lncRNAs is an important determinant of their functional mechanisms [51,52]. Cytoplasmic localization is compatible with a potential ceRNA-like mechanism, in which lncRNAs interact with miRNAs and influence miRNA-mediated repression of target mRNAs [53]. In the present study, 143 lncRNAs were predicted as potential ace-miR-750-y-interacting candidates (Figure 4(A)). Three candidates were predicted to localize to the cytoplasm, providing a basis for selecting lncRNA6470 for experimental validation.

FISH analysis showed overlapping cytoplasmic signals of lncRNA6470 and ace-miR-750-y (Figure 4(F-K)), supporting their spatial proximity within larval gut cells. Together with the dual-luciferase reporter assay (Figure 4(E)), these data provide additional support for a potential interaction between lncRNA6470 and ace-miR-750-y. Furthermore, expression profiling demonstrated an opposite expression trend between lncRNA6470 and ace-miR-750-y in 6-day-old larvae (Figure 1(D), 5(A)). Taken together, these findings support a binding relationship between lncRNA6470 and ace-miR-750-y and suggest that lncRNA6470 may negatively regulate ace-miR-750-y.

### LncRNA6470 participates in modulating larval survival, host immune response, and expression of selected A. apis genes

In current work, siRNA-mediated silencing of lncRNA6470 led to significant upregulation of ace-miR-750-y and significant downregulation of *AcPP2A* in the 6-day-old larval gut (Figure 5(C–E)). This suggests that lncRNA6470 may act upstream of the ace-miR-750-y/*AcPP2A* interaction, particularly at the late infection stage.

Following *A. apis* inoculation, silencing of lncRNA6470 significantly reduced larval survival (Figure 5(F)), indicating that lncRNA6470 may contribute to host survival during infection. By comparison, lncRNA6470 silencing did not significantly alter chalkbrood incidence (Figure 5(G)). The discrepancy between reduced survival and unchanged chalkbrood incidence demonstrates that lncRNA6470 may affect larval tolerance or physiological resilience during infection rather than simply determining visible disease incidence (Figure 5(F,G)). Zhou et al. [54] reported that certain lncRNAs in *Drosophila* can modulate immune homeostasis downstream of antimicrobial peptide pathways. In summary, these data suggest that lncRNA-regulated immune modulation or tissue-maintenance processes may contribute to host tolerance during *A. apis* infection.

In addition, lncRNA6470 silencing changed the expression of host immune genes and selected *A. apis* genes (Figure 5(H,I)). This indicates that lncRNA6470 affects both host immune status and pathogen-associated transcriptional responses. Because these immune and fungal genes were not validated as direct targets of lncRNA6470, their altered expression is more likely to reflect downstream or indirect effects of lncRNA6470 silencing.

### Rescue assay supports lncRNA6470/ace-miR-750-y/AcPP2A regulatory axis

Notably, this aligns with ceRNA theory in which lncRNAs modulate miRNA availability, thereby affecting downstream mRNA targets. Dual interference abolished the significant upregulation of *AcPP2A* triggered by miRNA knockdown, returning its expression to non-significant levels ((Figure 2(D), 6(A)). These results support a ceRNA model in which lncRNA6470 may influence *AcPP2A* expression through ace-miR-750-y, particularly at the late infection stage.

In the rescue assay, simultaneous lncRNA6470 silencing and ace-miR-750-y inhibition did not significantly affect larval survival compared with the control treatment, but it significantly increased chalkbrood incidence (Figure 6(B,C)). The incomplete rescue of larval survival and the increased chalkbrood incidence observed in our co-inhibition assay indicate that the disease phenotype is influenced not only by the lncRNA6470/ace-miR-750-y/*AcPP2A* axis, but also likely by additional parallel immune-regulatory pathways. Compared with lncRNA6470 silencing alone, dual interference produced a stronger suppressive effect on immune-gene expression in 6-day-old larval guts, where *Dorsal*, *Defensin*, *Hymenoptaecin*, and *PPO* were all downregulated (Figure 5(H), 6(D)). This pattern suggests that lncRNA6470 and ace-miR-750-y jointly regulate immune homeostasis, but it cannot be fully explained by miRNA sequestration alone. Dual interference may have disrupted the balance of the lncRNA6470/ace-miR-750-y/*AcPP2A* module and its associated immune responses. Because *A. apis* infection is countered in part by antimicrobial peptide- and melanization-related responses, suppression of these immune genes may contribute to the increased chalkbrood incidence observed after dual interference. Moreover, the expression of host immune genes was strongly inhibited after dual interference despite the absence of significant *AcPP2A* changes, showing that immune regulation by this module may involve *AcPP2A*-independent downstream effects (Figure 6(A,D)) [,^55^].

Taken together, our findings suggest that lncRNA6470 interacts with ace-miR-750-y and participates in the regulation of *AcPP2A* expression and host immune responses during *A. apis* infection. This regulatory module is associated with larval survival and chalkbrood susceptibility, providing new insights into ncRNA-mediated host–pathogen interactions in *A. cerana* larvae.

## Supplementary Material

Supplementary Table.docx

## Data Availability

The datasets generated during this study are available at Mendeley Data: https://data.mendeley.com/datasets/8x3y24mpxx/1 [56].
