# Supplementary material for: LncRNA6470/ace-miR-750-y axis modulates AcPP2A gene, immune response and survival of eastern honey bee larvae during fungal infection
Source: Virulence. 2026 Aug 2;17(1):2697761. doi: 10.1080/21505594.2026.2697761 (PMC13432886; doi:10.1080/21505594.2026.2697761)
Supplement: Supplementary Table.docx [file KVIR_A_2697761_SM1512.docx]

Supplementary Table

| miR-750-y-loop | CTCAACTGGTGTCGTGGAGTCGGCAATTCAGTT |
| --- | --- |
| miR-750-y-F | GCCGAGCCAGATCTAACTCT |
| miR-750-y-R | CTCAACTGGTGTCGTGGA |
| Mimic-miR-750 | CCAGAUCUAACUCUUCCAGCUCA |
|  | AGCUGGAAGAGUUAGAUCUGGUU |
| Mimic-NC | UUCUCCGAACGUGUCACGUTT |
|  | ACGUGACACGUUCGGAGAATT |
| Inhibitor-miR-750 | UGAGCUGGAAGAGUUAGAUCUGG |
| Inhibitor-NC | CAGUACUUUUGUGUAGUACAA |
| *U6 snRNA*-F | CCAGGAGATGAAGTGGATACTC |
| *U6 snRNA*-R | CTTGCTTGAACTGCTGCTT |
| *AcPP*2*A*-F | CTGCTTGCTTGATATGTGAG |
| *AcPP*2*A* -R | CGATGATACGACCAGTTGT |
| Ace-*actin*-F | TTATATGCCAACACTGTCCTTT |
| Ace-*actin*-R | AGAATTGATCCACCAATCCA |
| *AcPP*2*A*-m-F | AGCCAACTAATATGGAGGAG |
| *AcPP*2*A* -m-R | TGGATCTTCTGGTTCTTCAA |
| *AcPP*2*A*-m-wt-F | TTGACTGCTCGAAGAGAATT |
| *AcPP*2*A*-m-wt-R | AATCAGACCGCCGTATCT |
| *AcPP*2*A*-m-mut-F | CATGTCGTAGTCGGGTTGGAGGCCGTGACCCTCTGAGCTGTGCACTATC |
| *AcPP*2*A*-m-mut-R | TCGAGATAGTGCACAGCTCAGAGGGTCACGGCCTCCAACCCGACTACGACATGAGCT |
| *PPO*-F | CCTGAAGGTGCCAGAATT |
| *PPO*-R | CCAATGCCAATGATGAAGAT |
| *STE11-like-*F | TCCTACTGCTACACCTGAC |
| *STE11-like-*R | TAGAATACTGAGTGGTTGAGAG |
| *aflR-*F | CGTTGTCATAGGATAGGTAAG |
| *aflR-*R | TCTCAGATTCAGTTGCTTCA |
| *ski3-*F | TGGAGAAGAGGAACGAGAT |
| *ski3-*R | CGCTGTCATTGTCAAGGA |
| *5.8s rRNA-*F | GGCACTTTGTTAGGCTTTG |
| *5.8s rRNA-*R | GTTTGGTGACTCTTCTTCCTTC |
| lncRNA6470-F | GAAGGCAGAAGGAAGGTAG |
| lncRNA6470-R | GGTGATGGTGGTGGTAAC |
| lncRNA6470-m-F | TCTAGTTGTTTAAACGAGCTCCTCTTTTCTTCTCTCG |
| lncRNA6470-m-R | CAGGTCGACTCTAGACTCGAGCTCTTTTCTTCTCTCG |
| siRNA-lnc6470 | GGAUAAAGACCGAGGACGUTT |
|  | ACGUCCUCGGUCUUUAUCCTT |
| *Dorsal-*F | TGGATTCAATGTAGCAGGAT |
| *Dorsal*-R | CGTTCTCGTAAGTAGTAATGG |
| *Defensin*-F | AGCATCCTGAGAACGAAGA |
| *Defensin*-R | CGCAAGCACTGTCATTGA |
| *Hymenoptaecin*-F | CCATCGTCATTCAAGGAAC |
| *Hymenoptaecin*-R | TACTCCACCATAGGCATCT |
| *PPO*-F | CCTGAAGGTGCCAGAATT |
| *PPO*-R | CCAATGCCAATGATGAAGAT |
| ace-miR-750-y FISH Probe | TGAGC+TGGAAGAGT+TAGATCTGG |
| Lnc6470 FISH Probe | CTC+TGTC+TCCATCCCTCCCTA |
